# A Review of Machine Learning and Deep Learning Approaches on Mental Health Diagnosis

**DOI:** 10.3390/healthcare11030285

**Published:** 2023-01-17

**Authors:** Ngumimi Karen Iyortsuun, Soo-Hyung Kim, Min Jhon, Hyung-Jeong Yang, Sudarshan Pant

**Affiliations:** 1 Department of Artificial Intelligence Convergence, Chonnam National University, Gwangju 61186, Republic of Korea; 2 Department of Psychiatry, Chonnam National University Hwasun Hospital, Gwangju 58128, Republic of Korea

**Keywords:** machine learning, deep learning, mental health conditions, healthcare, mental health diagnoses

## Abstract

Combating mental illnesses such as depression and anxiety has become a global concern. As a result of the necessity for finding effective ways to battle these problems, machine learning approaches have been included in healthcare systems for the diagnosis and probable prediction of the treatment outcomes of mental health conditions. With the growing interest in machine and deep learning methods, analysis of existing work to guide future research directions is necessary. In this study, 33 articles on the diagnosis of schizophrenia, depression, anxiety, bipolar disorder, post-traumatic stress disorder (PTSD), anorexia nervosa, and attention deficit hyperactivity disorder (ADHD) were retrieved from various search databases using the preferred reporting items for systematic reviews and meta-analysis (PRISMA) review methodology. These publications were chosen based on their use of machine learning and deep learning technologies, individually assessed, and their recommended methodologies were then classified into the various disorders included in this study. In addition, the difficulties encountered by the researchers are discussed, and a list of some public datasets is provided.

## 1. Introduction

Mental health can be seen as a person’s emotional, psychological, and social well-being. It can be harmed by various mental health conditions, which negatively influence a person’s intellectual capacity, emotions, and social relationships. To combat these disorders, appropriate and timely assessment is essential to identify (diagnose) one from the other. The screening of mental health conditions is performed using self-report questionnaires designed to detect certain sensations or attitudes toward social interactions [1].

Machine learning (ML) is a subfield of artificial intelligence (AI) that deals with three problems: classification, regression, and clustering. It utilizes data and algorithms to mimic how people learn while progressively improving accuracy in various tasks [2]. ML has been applied to multiple areas of psychological treatments and offers excellent potential for predicting and treating mental health conditions and analogous health outcomes. Typically, these algorithms require significant data to learn patterns and perform classification tasks. One of the most widely applied ML approaches in the prediction of mental illnesses is supervised learning.

Supervised learning is the process of learning a mapping of a collection of input variables and an output variable and applying this mapping to predict the outcomes of unseen data [3]. Support vector machine (SVM) is a good example of supervised learning that deals with classification and regression problems. This method works based on the concept of margin calculation by finding the optimal decision line or boundary called the hyperplane to separate n-dimensional space into different classes. This involves placing new data points into the correct categories in the future. Some advantages of SVM include its ability to handle both semi-structured and structured data. Additionally, because it adopts generalization, there is a lower probability of overfitting. However, SVM also has some disadvantages. With large datasets, there is an increase in training time. Therefore, its performance begins to dwindle. Additionally, SVM does not work well on a noisy dataset. Decision trees are also supervised learning methods for classification and regression problems. A tree can be seen as a piecewise constant approximation. It creates models that predict the value of target variables by learning simple decision rules inferred from data features. Logistic regression predicts the output of a categorical dependent variable; therefore, its outcome can either be Yes or No, 0 or 1, etc. Finally, naïve Bayes uses the Bayes theorem of probability for classification. It assumes that a particular feature is unrelated to other features in a given dataset.

Another widely used ML approach is ensemble learning. It involves training several individual learners to solve a problem. This method creates multiple learners and combines them to form a single model, and each learner works as an individual traditional ML method. Ensemble learning comprises three classes, bagging, boosting, and stacking. Bagging creates multiple datasets through random sampling, builds multiple learners in parallel, and combines all the learners using an average or majority vote strategy. Boosting creates multiple datasets through random sampling with replacement overweighted data and builds learners sequentially. These learners are then combined using a weighted averaging strategy. Stacking, on the other hand, either begins with bagging or boosting, and the outputs of the learners serve as inputs to another traditional ML algorithm (meta-model). The meta-model then acts as an aggregate by combining the outputs to provide results. Random Forest (RF) and extreme gradient boosting (XGBoost) are some of the most widely used ensemble learning methods. Random forest uses the bagging method to create decision trees with subsets of data, and each decision tree’s output is combined to make a final decision tree. XGBoost, on the other hand, is a scalable distributed gradient-boosting method of the decision tree.

Transfer learning is another ML method that researchers in this area are exploring. In simple terms, it is the transfer of knowledge from a related task that has already been learned to improve learning in a new task [4]. Although these algorithms open up new avenues for psychological research [5], their widespread use raises ethical and legal concerns about data anonymization.

ML is divided into various subfields, one of which is deep learning (DL) [6]. DL is a branch of ML that can take unstructured data such as text and images in its raw form and automatically finds the set of characteristics that differentiate distinct categories of data. Hence, one does not need to identify features as the architecture learns these features and increments on its own; therefore, it requires the utilization of a more extensive amount of data. Recently, there has been much interest in developing DL for mental illness diagnosis.

Since the introduction of AI into the medical sector, numerous studies and publications have been conducted on the use of ML and DL to intensify the examination of different medical problems. The application of AI in the medical sector has also extended to mental health condition diagnosis due to its great importance [7]. A number of advancements have been made in the application of ML for the diagnosis of mental health conditions. Integration with electronic health records (EHRs) is one such advancement. It is a growing trend in analyzing data from EHRs to assist with diagnosing mental health conditions. ML algorithms are also trained to analyze data retrieved from wearable devices such as smartwatches and fitness trackers. This approach has the potential to provide continuous monitoring of mental health status and enable the early detection of potential issues. Additionally, ML is applied in predictive modeling. Here, ML algorithms can identify individuals at risk of developing mental health conditions. This can allow for early intervention and prevent more severe mental health issues. Finally, ML is applied in the development of automated screening tools to help identify individuals at risk for certain mental health conditions.

ML is used to identify mental health conditions by analyzing patterns in data indicative of certain conditions. These data can be generated and collected from various sources, such as patient records, brain imaging scans, or even social media posts. For this purpose, different algorithms are used, including supervised learning algorithms, which are trained on labeled data, and unsupervised learning algorithms, which can identify patterns in data without the need for explicit labels. Once a model has been trained on the collected dataset, it can then be used to predict the likelihood that an individual has a particular mental health condition based on their data. ML researchers perform this prediction by applying the learned patterns to new data and using the models’ output to make a diagnosis.

Research in this area has been carried out using various ML techniques, and recently has been noted to extend to DL. In [8], Shamshirband et al. examined the use of convolutional neural networks (CNN), deep belief networks (DBN), auto-encoders (AE), and recurrent neural networks (RNN) in healthcare systems. They addressed several concerns and challenges with DL models in healthcare, as well as significant insights into the accuracy and applicability of DL models. In another review [9], the authors focused on previous studies on ML to predict general mental health problems and proposed possible future avenues for investigation.

Librenza-Garcia et al. [10] reviewed past studies on diagnosing bipolar disorder patients through ML techniques. He et al. [11] surveyed automatic depression estimation (ADE) methods relating to deep neural networks (DNN) and presented architectures based on audio-visual cues. Finally, in a review of PTSD, Ramos-Lima [12] reviewed the use of ML techniques in assessing subjects with PTSD and acute stress disorder (ASD).

This study aims to contribute to mental health condition diagnosis research with the use of machine learning techniques in the form of a systematic study. It examines the developments in ML and DL to provide a summary of the methods used by researchers over the last decade to diagnose or predict the most common mental health conditions affecting our communities today. This study further draws attention to the challenges and limitations researchers face in this area and discusses possible opportunities for future research; a list of publicly available datasets is also provided. This review hopes to assist practitioners attempting to enhance mental health condition diagnosis and treatment through AI by providing them with a summary of the techniques used in the last decade. Hence, the articles reviewed for this study were obtained from credible sources, evaluated, summarized, and classified into seven mental disorders.

The rest of the paper is organized as follows: the review background gives a general understanding of all the mental health problems included in this study; the method of data selection, extraction, and analysis discusses the method used for data selection; ML and DL methodologies dive deep into the various methodologies applied by researchers in the articles reviewed; the analysis and discussion focus on the detailed analysis of the study and challenges; and the final section provides the conclusion of this paper.

## 2. Review Background 

According to the World Health Organization (WHO), in 2019, anxiety and depression were the most common mental health conditions among the estimated 970 million people worldwide living with mental health problems. However, this number rose remarkably due to the onset of the COVID-19 pandemic in 2020. With this pandemic grew the importance of gaining access to medical care and treating mental illnesses. Although these options exist, most people do not gain access to them, and many of them face discrimination, stigma, and violation of their human rights [13].

Diagnosing mental health issues involves a thorough psychiatric interview, usually covering the suspected symptoms, psychiatric history, and physical examinations. Psychological tests and assessment tools are also helpful when identifying psychiatric symptoms [14].

Various research has been carried out on mental illness diagnoses. For this review, we focused our search on schizophrenia, depression, anxiety, bipolar disorder, post-traumatic stress disorder (PTSD), anorexia nervosa, and attention deficit hyperactivity disorder (ADHD) concerning ML and DL.

Schizophrenia is a severe mental illness that affects a person’s ability to interpret reality, thus causing an abnormal interpretation of reality. A report by the World Health Organization stated that schizophrenia affects at least 1 in 300 people worldwide. Additionally, it increases the likeliness of death of patients by about two to three times due to their proneness to cardiovascular, metabolic, and infectious diseases [15]. It may result in delusions, hallucinations, disorganized speech, disorganized behavior, and negative symptoms. This may ultimately lead to social and occupational dysfunction [16].

Depression (major depressive disorder) is one of the widespread mental illness commonly screened through the Patient Health Questionnaire (PHQ) [17]. It is usually identified through symptoms of deep sadness and loss of interest in activities, leading to a decrease in a person’s ability to function correctly. Shorey et al. [18], in their study, stated that 34% of adolescents between the ages of 10 and 19 are at risk of clinical depression, exceeding the estimates of individuals aged between 18 and 25. Their study also showed that the Middle East, Africa, and Asia have the highest prevalence of elevated depressive symptoms; however, female adolescents reportedly have a higher prevalence of elevated depressive symptoms than male adolescents. Depression, if not properly attended to, may result in suicidal ideations and suicide [19].

Anxiety brings about the feeling of worry or fear that can be mild or severe. Anxiety on its own is a symptom of several other conditions, such as social anxiety disorder (social phobia), panic disorder, and phobias. Although everyone feels some anxiety at some point, it becomes a problem to be taken into serious consideration when they find it hard to control these feelings when they constantly affect their daily lives. Some general anxiety symptoms include dizziness or heart palpitations, trouble sleeping, a lack of concentration, restlessness, and worry. It is estimated that about 264 million people suffer from anxiety disorder, and a study conducted in 2020 showed that 62% of respondents to a survey reported some degree of anxiety, and a higher percentage of those affected by this disorder are women [20].

Another form of mental disorder is called bipolar disorder, formally known as “manic-depressive illness” or “manic-depression,” which causes an unusual change in mood, a reduction in energy, and lower activity levels and concentration levels. The stated mood changes range from periods of extreme highs (manic episodes) to lows (depressive episodes), as well as less severe episodes (hypomanic episodes). Studies show that about 46 million people worldwide present with bipolar disorder at different levels. People with this disorder may also be at risk of suicide, with about 60% showing signs of substance misuse [21]. It is usually diagnosed at different points in a person’s life based on symptoms, life history, experiences, and on rare occasions, family history.

Flashbacks, nightmares, and severe anxiety characterize PTSD, as well as constant uncontrollable thoughts triggered by terrifying events that a person either experienced or witnessed. To properly diagnose PTSD, medical personnel perform physical examinations on the suspected patient to check for medical issues that may have caused the prevailing symptoms. They conduct a psychological evaluation to discuss the events that might have triggered the appearance of the symptoms and use the criteria in the Diagnostic and Statistical Manual of Mental Disorders (DSM-5) to diagnose the illness efficiently [13]. Like most mental illnesses, PTSD is also not curable, but can be managed with proper treatment (mostly psychotherapy), which can help an affected person gain control over their life. With a lifetime prevalence of 8% in adolescents between the ages of 13 and 18, statistics also show that about 3.5% of U.S. adults report cases of PTSD yearly [22].

Anorexia nervosa is a life-threatening eating disorder with no fully recognized etiology that affects people of all ages, regardless of gender. Statistics show that about 9% of the population worldwide suffer from eating disorders and about 26% of those affected are at risk of suicide attempts [23]. Over the years, anorexia has become the most dangerous mental health condition among young women and girls in well-developed societies [24]. The affected people place extreme importance on controlling their body weight and shape using severe methods. They are usually never satisfied with their body weight, no matter how much weight they lose. They may drastically control their calorie intake by throwing up after eating or misusing laxatives, diuretics, or enemas. They may also exercise excessively to lose weight [25].

Finally, when considering neurodevelopmental disorders, two major conditions stand out: autism spectrum disorder (ASD) and attention deficit hyperactivity disorder (ADHD). ASD impacts development and involves a broad range of conditions characterized by challenges with social skills, restricted and repetitive behaviors, and deficits in speech and nonverbal communication. ASD and ADHD are both neurodevelopmental disorders that tend to have neurological effects on the functioning of the nervous system, with similar symptoms. Often, one disorder is confused with the other. For this review, we focused our study on today’s most common mental health conditions. Therefore, we only included ADHD because it is the most common mental health condition around the world. Our search found that ADHD affects about 9% of children between the ages of 3 and 17 and at least 4–5% of adults in the US, as compared with ASD, which affects just 1–2% of people across North America, Asia, and Europe [26]. Children with ADHD usually have a lot of trouble focusing. They tend to be overly active and act without control over their impulses. ADHD is a mental illness that runs in families and is hard to be cured, although it can be managed if diagnosed earlier in the child’s life. Several steps are involved in diagnosing ADHD. The suspected patient must have shown about six or more inattentiveness, hyperactivity, and impulsiveness symptoms. The family history of the suspected patient is also taken into consideration. ADHD is treated with a combination of medication and behavior therapy.

## 3. Method of Data Selection, Extraction, and Analysis

For this review, several thoughts and questions were considered in the selection, extraction, and analysis of past research to provide an overview of the trends in mental illness diagnosis research. Questions such as “what are the recent methods used by ML researchers for mental illness diagnosis over the years?” and thoughts on the challenges faced by these researchers were considered. Moreover, we sought to put together a list of accessible datasets which can serve as a knowledge base for ML researchers.

**Figure 1:**
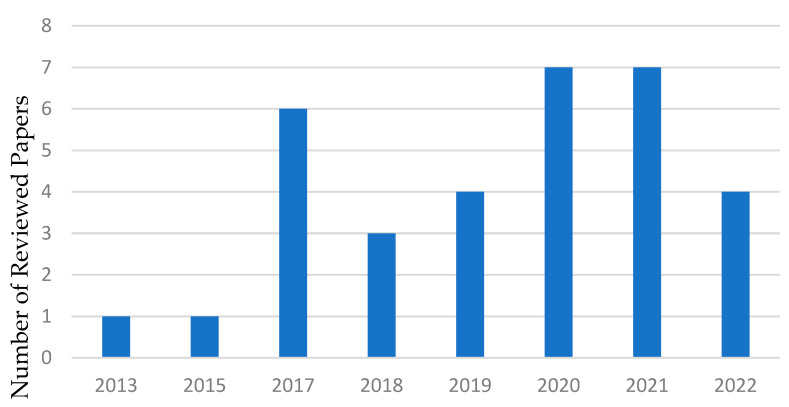
The proportion of reviewed articles included in this study by year with 2020 and 2021 having the most papers.

**Figure 2:**
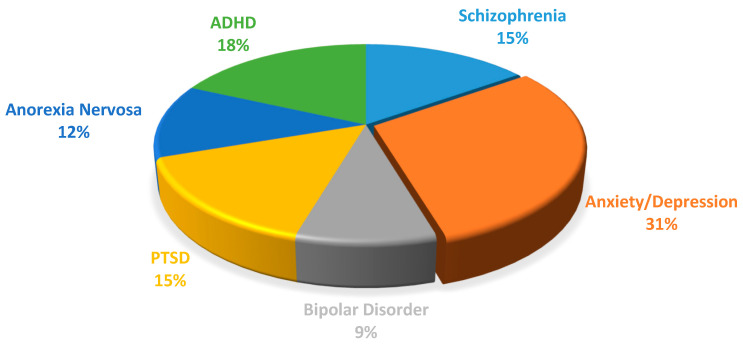
Percentage of all articles for each mental disorder included in this study.

We researched search-related databases between 2013 and 2022, including Google Scholar, PubMed, Scopus, and Web of Science, using search terms such as “Artificial Intelligence for mental health diagnosis” and “Machine Learning for mental health prediction”. Figure 1 shows a chart of the number of articles included in this review from 2013 to 2022, with 2020 and 2021 having the most papers. Figure 2 depicts the percentage of each mental health condition considered in this study. The processing of results was conducted based on a reversed version of the Quality of Reporting of Meta-analyses (QUOROM Statement) [27], called the Preferred Reporting Items for Systematic Reviews and Meta-Analyses (PRISMA) guidelines [28], which addresses several conceptual and practical advances in the science of systematic reviews.

**Figure 3:**
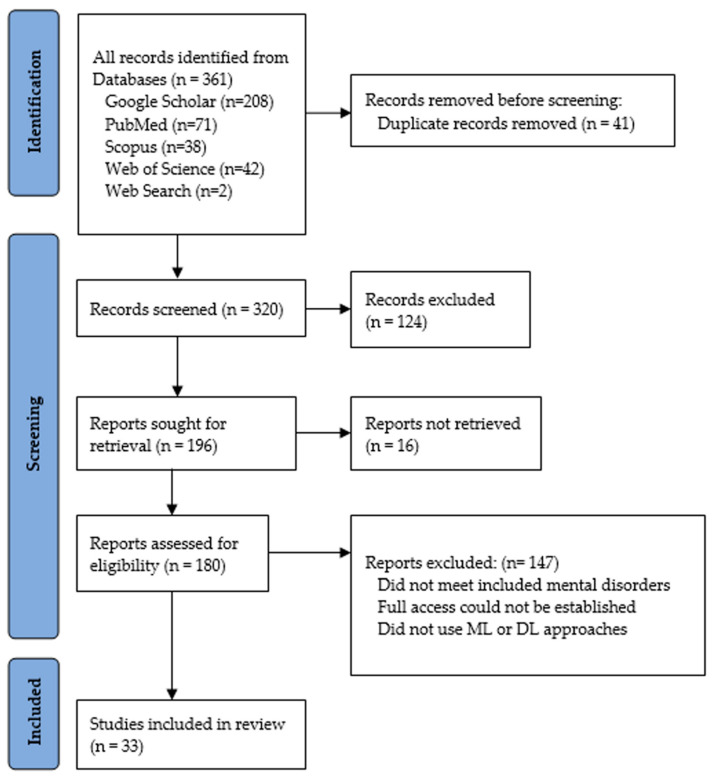
PRISMA flow diagram of the study selection process.

Figure 3 shows a detailed representation of the PRISMA flow diagram used in the literature selection and inclusion in this review. All publications were carefully selected and evaluated, and the articles which did not meet the selection criteria were excluded if:

1. the research did not examine at least one of the mental health issues included in this study,
2. full access to the article could not be established, and
3. the proposed approach did not use an ML or DL approach.

## 4. ML and DL Methodologies Applied

Different approaches or methods are applied to predict mental illnesses. In AI, these methods include ML, DNNs, and even robotics. These approaches primarily aim to find causes, diagnose, and predict treatment outcomes of these disorders. Using the Open Source Mental Illness (OSMI) survey from 2019, [29] proposed a method to find the features that negatively influence the mental health of employees in technical and non-technical companies and also predict the mental health condition of these employees. In another study, Katarya et al. used ML technologies to identify predictors of psychological distress during the COVID-19 pandemic [30]. Their study showed the need for measuring an individual’s physical experience of psychological distress and emotion control mechanisms to assist mental health clinicians in tailoring evaluations and treatment amid a global health crisis.

### 4.1. Approaches for Schizophrenia Prediction

Srinivasagopalan et al. [31] proposed a method to automatically diagnose patients with schizophrenia by using structural and functional magnetic resonance imaging (fMRI) modalities from brain scans. The proposed method was trained on data collected from the Mind Research Network and tested on traditional ML algorithms such as logistic regression (LR), support vector machine (SVM), and random forest (RF). Then, they applied feature selection to improve the model accuracy. Furthermore, they used a DL binary classifier with three hidden layers. Their results showed that the DL approach showed higher accuracy than the traditional ML methods for diagnosing schizophrenia.

Another approach for the prediction of schizophrenia has been proposed by Zeng et al. [32]. They focused on the automatic diagnosis of schizophrenia using a Discriminant Autoencoder Network with Sparsity constraint (DANS) to learn imaging site-shared functional connectivity functions on a dataset comprising 1000 participants. Their proposed DANS method showed that 85% accuracy was obtained from multi-site pooling classification and 81% accuracy from leave-site-out transfer classification. Thus, this proved that their method could learn connectome patterns, aid the study of pathophysiology, and obtain accurate schizophrenia prediction across numerous independent imaging locations.

**Figure 4:**
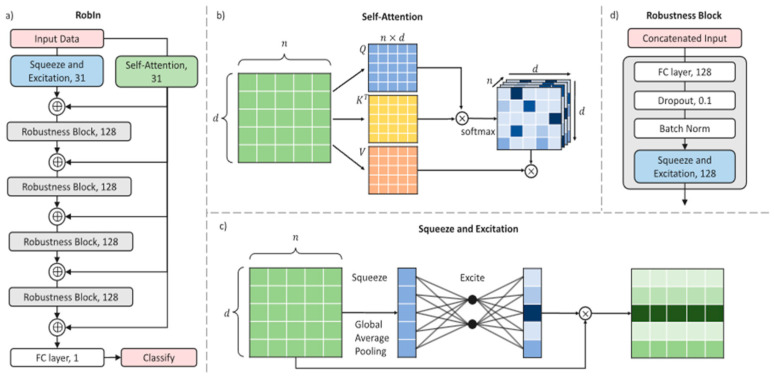
Structure of Robin architecture implemented by Organisciak et al. [33]. (**a**) An overview of the entire network with output dimensions of each layer represented. Input data goes down the robustness stream and the interpretability stream; (**b**) self-attention mechanism: the input representation is converted into a key, query and value matrix, the cosine distance between each query and each key is found via a matrix multiplication with a higher activation signalling higher alignment between query and key; (**c**) squeeze and excitation: each attribute *i* = 1, …, *d* is squeezed down to a representative number, a miniature neural network excites the squeezed information to evaluate how important each attribute is, then the initial data is multiplied by the importance scores; (**d**) the robustness block we propose in this paper.

To facilitate actual world usage and bridge the large training application gap, Organisciak et al. [33] in their paper developed a robust interpretable framework by combining the “squeeze and excitation” and “self-attention” complementary attention mechanisms to provide insights into the network’s decision process (Figure 4). Data for the experiment were collected from the Lagos University Teaching Hospital, Lagos, Nigeria, and contained a total of 151 subjects. Due to the small amount of data, they used the validation protocol by experimenting on the standard 90/10 cross-validation and a 50/50 train/test split with 25 runs. Their research found that the proposed method (RobIn) outperformed other methods that used both test settings with a 98% accuracy and 98.56% F1-Score on the 90/10 cross-validation test and 86.45% on accuracy on the 50/50 train/test split.

Birnbaum et al. [34] proposed a human-to-computer approach by combining social media content with clinical appraisals to explore social media as a tool to identify individuals with schizophrenia. Data were collected from publicly available Twitter posts from 2012 to 2016 with a self-disclosed diagnosis of schizophrenia using a Twitter crawler called GetOldTweetsAPI. They modeled a classifier on 10-fold cross-validation. The results showed that the resultant classifier discriminated actual schizophrenia disclosures from control users with a mean accuracy of 0.88% and best performance of 0.95 by the same AUC metric.

In the classification of schizophrenic patients from non-schizophrenic patients, Jo et al. [35] proposed the use of network analysis for this task. The data were estimated on a number of global and nodal network properties from graphs reconstructed using probabilistic brain tractography. The authors then built various ML models based on their proposed brain network properties.

### 4.2. Approaches for Depression and Anxiety Detection

Anxiety prediction is one of the trickiest mental illness predictions based on its similarities to major depressive disorder (MDD) in a clinical framework [36]. In [37], Sau et al. used ML methods to predict depression and anxiety in geriatric patients. A total of 10 different classifiers were tested on a selected set of features, and an accuracy of 89% was achieved with RF (RF). Meanwhile, in [38], Sua. A. et al. went further to predict anxiety and depression in seafarers through the hospital anxiety and depression scale. Five different ML classifiers were tested. In this case, Catboost provided the best result, with an accuracy of 82.6% and a precision of 84.1%, compared to RF, with both an accuracy and precision of 81.2%.

Niu et al. [39] used a DL model for automatic depression detection using the hierarchical structure of depression detection and graph attention network. The experiment was conducted on the DAIC-WOZ dataset [40]. The result showed an F1-Score, mean absolute error (MAE), and root mean square error (RMSE) of 0.92, 2.94, and 3.80, respectively. Another attempt at depression detection was conducted by Yoon et al. [41]. They developed a multimodal DL model for depression detection in their paper. The data used for their experiment were from a self-acquired multimodal dataset consisting of 961 vlogs from YouTube. Their result showed 65.40% precision, 65.57% Recall, and a 63.50 F1-Score compared to baseline models. In another study by Xezonaki et al. [42], a Hierarchical Attention Network was used to classify interviews with depressed patients because these sessions are made up of turns and words, thus proving a hierarchical textual structure. The study was conducted on the General Psychotherapy Corpus and the DAIC-WOZ depression dataset and achieved 71.6 and 68.6 F1 scores, respectively.

Cho et al. [43] proposed depression detection using a database collected from the medical checkup cohort of the National Health Insurance Sharing Service (2019) in Korea using an RF algorithm. In their study, 0.02% of the participants had depression, and 99.8% were not depressive; this gave reason for applying down-sampling or up-sampling to ensure a balance between the two groups. At the end of the study, the authors acquired an AUC of 0.849. Meanwhile, in an attempt to enhance the detection of depression, Sharma et al. [44] in their paper proposed an ML method using a Lifelines Database containing biomarker data and self-reported depression data. The dataset used in this study also had imbalanced data; therefore, different resampling approaches were implemented to address this issue. An extreme gradient boosting (XGBoost) algorithm was applied to each sample.

Supervised learning [45] is implemented in a lot of research to predict mental illnesses. One such case is the paper by Deshpande et al. [46]. Their study implemented naïve Bayes and SVM classifiers on Emotion AI to collect and preprocess textual data. Their result showed that the multinomial naïve Bayes classifier performed better than the SVM classifier. In [47], Hilbert et al. also used supervised learning based on an SVM on multimodal biobehavioral data to separate subjects of anxiety from subjects of depression. For this study, clinical questionnaire data, cortisol release, structural brain data, gray matter (GM), and white matter (WM) volumes were used separately and in combination. Their study showed that the use of clinical questionnaires alone for the classification of anxiety proved to be complicated. However, the use of cortisol and gray matter volume data, on the other hand, provided incremental benefit for anxiety categorization.

To provide a novel and objective diagnostic tool for anxiety and depression differentiation, Richter et al. [48] presented the use of cognitive behavioral performance data incorporated with ML. In their study, questionnaires were assigned to subclinical participants belonging to four major symptom groups—a high level of anxiety, a high level of depression, a high level of anxiety and depression, and the controls (low anxiety and depression symptoms). Their cognitive behaviors were measured using a battery of six different cognitive behavioral tasks to access various biases. The data were then analyzed using an RF algorithm, and the model strictly assigned participants based on their aggregated cognitive performance.

### 4.3. Approaches for Bipolar Disorder Detection

Many studies have been conducted on detecting bipolar disorder using single-modality MRI images. However, Li et al. [49] proposed using multimodal MRI data. They designed an SVM model with voxel-based morphometry (VBM) for focal differences in brain anatomy to achieve this. They also used regional homogeneity (ReHo) for evaluating brain activity to discriminate patients with bipolar disorder from controls. Their study showed that the fusion of structural and functional MRI could greatly support diagnosing bipolar disorder.

**Figure 5:**
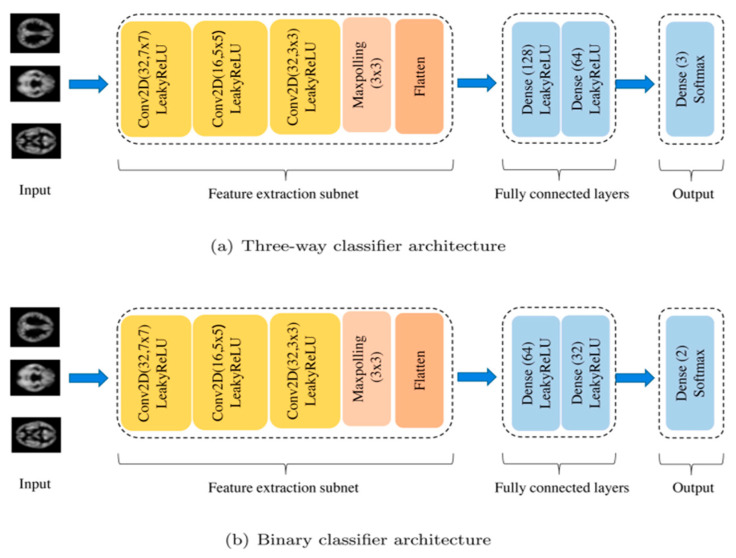
Schematic of end-to-end CNN pipeline. (**a**) The architecture of three-way classifier. (**b**) The architecture of binary classifier. They have the same feature extraction subnet. The difference between them is just the number of hidden neurons in the fully connected layer [50].

Researchers in this area have also implemented some DL approaches. One such study was carried out by Li et al. [50]. They proposed the use of a CNN on structural magnetic imaging (sMRI) data (Figure 5) to automatically diagnose first-episode psychosis (FEP), bipolar disorder (BD), and healthy controls (HC). The CNN showed better performance when compared with a three-way classifier (FEP vs. BD vs. HC) and three binary classifiers (FEP vs. BD, FEP vs. HC, BD vs. HC).

In an attempt to categorize bipolar disorder from visual data into various clinical states with the use of a hybrid model, Abaei and Osman [51] applied CNN to extract non-verbal features from video recordings of the Turkish Audiovisual Bipolar Detection Corpus [52], popularly known to have been used in the AVEC 2018 Bipolar disorder and cross-cultural affect recognition challenge [53]. The extracted features are then passed into a long-short-term memory (LSTM) and are used to determine and classify the clinical state of bipolar.

### 4.4. Approaches for Post-Traumatic Stress Disorder (PTSD) Detection

To develop an accurate post-earthquake PTSD risk score that performs better than regression methods, Rosellini et al. [54] proposed using an ensemble model called the super learning algorithm. The study was conducted on respondents before and after the 8.8-magnitude earthquake in February 2010. With the super learning algorithm, the authors could predict the risk score of PTSD more accurately than with conventional regression methods. Schultebraucks et al. applied ML techniques to examine a sizeable multidimensional dataset of soldiers before being deployed to Afghanistan to predict PTSD [55]. The dataset comprised 473 army personnel of the 101st Airborne at Fort Campbell, Kentucky. The data were collected from clinical assessments, RF was used for predictive modeling, and SVM was used as a benchmark for comparison. Their study found that the clinical prediction of post-deployment symptom trajectories and provisional PTSD diagnosis obtained significant discrimination based on the pre-deployment data collected. Reece et al. [56] utilized textual data from 204 individual Twitter users and extracted 279,951 tweets carrying signs of depression to build a supervised learning model with different classifiers. The predicted results were later replicated and tested on 174 Twitter users with 243,775 tweets diagnosed with PTSD. Out of the several classifiers built, a 1200-tree RF classifier outperformed the others and was reported for both daily and weekly observations, as shown in Table 1 (MVR, DC, TBA, and NHC all indicate previous studies).

Based on the study conducted by Campbell et al. [57], decision tree analysis was used to predict unit-level risk for mental health outcomes in a Combat and Operational Stress Level Control (COSC) survey. The data used for the study consisted of 2290 officer and enlisted US Navy sailors, intact battalions, ground-based aviation squadrons, and medical support to the Marine Corps between 2007 and 2008 in Iraq and Afghanistan. The decision tree algorithm performed well in predicting high-risk PTSD on the validation set but mispredicted about 10% on independent samples.

As with other mental disorders that emerge from trauma, identifying mental illnesses that develop as a result of childhood sexual abuse is a challenge in clinical practice and research. In an attempt to determine the development of post-traumatic stress disorder (PTSD) and depression in sexually abused children, Gokten and Uyulan [58] researched children and adolescents who were sexually abused. After a forensic evaluation of each child, psychiatrists assessed the subjects based on the Diagnostic and Statistical Manual of Mental Disorders-Fifth Edition (DSM-V) diagnostic criteria, and a predictive model was developed using the RF classifier.

### 4.5. Approaches for Anorexia Nervosa Detection

A computer-aided therapeutic diagnosis based on sentiment analysis was implemented by Spinczyk et al. for the diagnosis of anorexia nervosa. The dataset used contained data from 44 anorexic and 52 healthy girls aged between 12 and 18. They proposed a method that dealt with the patients’ statements about their bodies, general sentiment analysis based on RNN, the intensity of their emotions, and sentiment analysis based on the dictionary approach [24]. Their study showed that the RNN method performed better, with 72% effectiveness in the diagnosis of anorexia.

To detect signs of anorexia nervosa from textual data, Paul et al. [59] used data from the CLEF eRisk 2018 challenge (Task 2) [60]. Different classification methods were used for this test to validate BOW features and UMLS features and also combine BOW and UMLS features, respectively. These methods include Ada Boost, LR, RF, and SVM. From their findings, the SVM proved to be better than other methods on the BOW features with a precision of 0.97, Recall of 0.98, and an F-measure of 0.98. On the performance on UMLS features, SVM showed higher performance in terms of the F-measure, at 0.55. However, with the combination of BOW and UMLS, the Ada boost classifier outperforms the other classifiers with an F-measure of 0.47.

In their paper, Guo et al. [61] used genome genotyping data containing 390 anorexia patients and 9266 non-anorexic patients to collect different sources for predicting the risk prediction of anorexia nervosa. The dataset was randomly split into the training and test set and trained on an LR model, SVM, and Gradient Boosting Trees for comparison. Meanwhile, using an extended version of the CLEF eRisk 2018 Task 2 challenge dataset [60], Ranganathan et al. [62] proposed the variational use of a Deep Learning RNN-LSTM and a Stochastic Gradient Descent Classification (SGDC) classifier for the possible early prediction of anorexia.

Finally, transfer learning was used to prove the possible diagnosis of anorexia nervosa using DNNs and transformer-based models based on Spanish tweets. The Spanish Anorexia Detection (SAD) dataset [63] was used for evaluating these models. No significant difference was recorded on the DNN model, as the CNN slightly outperformed the LSTM and BiLSTM, while on the transformer-based models, the BETO model performed best with an F1-Score of 94.1% [64].

### 4.6. Approaches for Attention Deficit Hyperactivity Disorder (ADHD) Detection

Mikolas et al. proposed a method for detecting patients with ADHD from a broad spectrum of other mental illnesses using anonymized clinical records [65]. The authors used an SVM classifier on 30 features, a secondary classification method without demographic characteristics (sex and age), and a secondary classification without missing data. They achieved 66.1%, 65.1%, and 68.8% accuracies, respectively.

In another study, Tan et al. [66] implemented a group-level mask on MRI images. The brain regions that appeared to be functioning during the fMRI imaging were measured and referred to as functional volumes. These were compared with regional brain volumes measured from anatomical images. The functional volumes performed better in classifying ADHD patients using an SVM classifier with functional volumes and demographic variables to obtain 67.7% balanced accuracy.

As stated earlier, ADHD can also be identified in adults. In this light, Tachmazidis et al. [67] carried out research on the diagnosis of ADHD in adults who underwent clinical diagnosis over time. Clinical data and questionnaires were used with a hybrid method consisting of an ML model, and a knowledge-based model was used for this experiment. Their approach showed an accuracy of 95% and has been deployed for testing in a clinical environment. Peng et al., proposed using an extreme learning machine (ELM) to diagnose ADHD [68] automatically. Features were extracted from a 110-participant dataset and evaluated on both ELM and SVM with leave-one-out cross-validation. ELM showed better performance than SVM in this case, with an accuracy of 90.18% compared with 86.55% forthe SVM.

In a study by Yin et al. [69], the use of a multi-site ADHD dataset comprising the ADHD-200 dataset of Peking University (PKU) and New York University (NYU) (Table 2), with 236 and 192 subjects, respectively, was used to determine if neural flexibility can serve as a biomarker to differentiate children with ADHD from typically developing children (TDC). They proposed the implementation of an extreme gradient boosting (XGBoost) algorithm to develop a classification model that can differentiate subjects with ADHD from TDC and a regression model to predict the ADHD severity of individuals. Their study found that neural flexibility is altered in children with ADHD and demonstrated the potential clinical utility of neural flexibility to identify children with ADHD and monitor treatment responses and disease severity (Table 3).

Finally, Liu et al. [70] presented the development of an algorithm based on convolutional denoising autoencoder (CDAE) and adaptive boosting decision Tree (AdaDT) to improve the classification of ADHD with the use of fMRI images. They extracted 3D spatial information from the selected ADHD-200 dataset using CDAE and reduced the extracted features using principal component analysis (PCA) to avoid over-fitting effectively. Their method showed an improvement in its classification performance of ADHD.

**Table 2:** Datasets for various mental health predictions.

No	Dataset	Application	Author	Data Type	Year
1	Distress Analysis Interview Corpus (DAIC) [40]	Anxiety, Depression, PTSD	Gratch et al.	Audio/Video	2014
2	Turkish Audio-visual Bipolar Disorder Corpus [52]	Bipolar Disorder	Çiftçi et al.	Audio/Video	2018
3	eRISK [60]	Anorexia Nervosa	CLEF	Text	2018
4	Spanish Anorexia Dataset (SAD) [63]	Anorexia Nervosa	López Úbeda et al.	Text	2019
5	ADHD-200 [71]	ADHD	The ADHD-200 consortium [72]	Images	2012
6	Danish Depression Database [73]	Depression	Videbech et al.	Audio/Video/Reported	2011
7	Reddit Self-reported Depression Diagnosis (RSDD) dataset [74]	Depression	MacAvaney et al.	Text	2017
8	Penn-dataset [75]	Schizophrenia	Hamm et al.	Video/Images	2014
9	AVEC 2013 Audio-visual Depressive Language corpus (AViD Corpus) [76]	Depression	Valstar et al.	Audio/Video	2013
10	AVEC 2014 [77]	Depression	Valstar et al.	Audio/Video	2014
11	AVEC 2016 [78]	Depression	Vasltar et al.	Audio/Video	2016
12	Crisis Text Line [79]	Depression	Lieberman and Meyer	Text	2013
13	DementiaBank Database [80]	Depression	Becker et al.	Audio/Video	1994
14	SemEval-2014 Task 7 [81]	Depression	Pradhan et al.	Text	2014
15	Emotional Audio-Textual Depression Corpus (EATD-Corpus) [82]	Depression	Shen et al.	Audio/Text (Chinese)	2022

**Table 3:** Summary of papers included in this review.

Author	Target	Data Domain	Data Size	Methodology	Model Performance	Model Validation	Motivation
Srinivasagopalan et al. [31] (2019)	Schizophrenia	Image (fMRI, sMRI)	144 subjects (75 controls 69 patients)	LR SVM RF NN (3 hidden layers)	Accuracy: LR: 82.77% SVM: 82.68% RF: 83.33% NN: 94.44%	CV	To automatically diagnose schizophrenia from brain MRI scans.
Zeng et al. [32] (2018)	Schizophrenia	Image (MRI.)	1000+ subjects 474 patients 607 controls	Deep discriminant auto-encoder network: Multi-site pooling classification, Leave-site-out transfer classification.	Accuracy: Multi-site pooling classification: 85.0% Leave-site-out transfer classification: 81.0%	10-fold CV Cross-site CV	To distinguish schizophrenia patients from healthy controls in a large multi-site sample.
Organisciak et al. [33] (2022)	Schizophrenia	Clinical observations data	97 patients 54 controls	Robust, interpretable framework based on Squeeze and Excitation and Self-Attention with 10-fold cross-validation.	Accuracy: 98%	10-fold CV	To improve the interpretability of DNNs for the diagnosis of schizophrenia.
Birnbaum et al. [34] (2017)	Schizophrenia	Text (Twitter)	671 users	Gaussian naïve Bayes RF LR SVMs	AUC Score: RF: 88.0%	10-fold CV	To accurately diagnose schizophrenic patients from noisy inference diagnosis.
Jo et al. [35] (2020)	Schizophrenia	Image	48 Schizophrenic 25 healthy controls	SVM Multinomial naïve Bayes RF XGBoost	ML model: Global RF: Accuracy: 68.0% AUC: 0.680 ML model: Four per Nodal network XGBoost: Accuracy: 66.3% AUC: 0.656	10-fold CV	To analyze brain network properties in patients with schizophrenia from healthy controls.
Sau and Bhakta [37] (2017)	Anxiety	Clinical data	510 geriatric patients	BN, logistic, multiple layer perceptron, NB, RF, random tree, J48, SMO, random subspace, KS	RF: Accuracy: 89%, TP rate: 89%, Precision: 89.1%, F-measure: 89%, AUC: 94.3% FP rate: 10.9	10-fold CV	Development of an automated predictive model for the prediction of anxiety in geriatric patients.
Sau and Bhakta [38] (2019)	Anxiety and Depression	Text (Interview based)	470 seafarers	Catboost LR Naïve Bayes RF SVM	Accuracy: Catboost: 89.3% LR: 87.5% Naïve Bayes: 82.1% RF: 78.6% SVM: 82.1%	10-fold CV	To detect depression in seafarers due to their susceptibility to mental health disorders.
Niu et al. [39] (2021)	Depression	Text and Audio	DAIC-WOZ dataset	Hierarchical Context-Aware Graph Attention Model	F1-Score: 0.92 M.A.E.: of 2.94 RMSE: 3.80	57%:19%:25% RS	To grasp sufficient logical and relational interview questions for automatic depression detection.
Yoon et al. [41] (2022)	Depression	Visual and Audio	961 YouTube Vlogs	Multimodal cross-attention mechanism	Precision: 65.40 Recall: 65.57 F1-Score: 63.50	70%:10%:20% RS	To detect depression from non-verbal behaviors.
Xezonaki et al. [42] (2020)	Depression	Text (Interview and therapy)	i. GPC 1,262 therapy sessions: 881 “not-depressed” 381 “depressed.” ii. DAIC-WOZ transcripts	Hierarchical attention networks	F1-Score: GPC dataset: 71.6 DAIC-WOZ: 68.6	5-fold CV	To predict depression levels with the use of data retrieved from psychotherapy sessions.
Cho et al. [43] (2020)	Depression	Clinical medical checkup data	433,190 subjects 10,824 depressed 422,364 non-depressed	RF	AUC: 0.849, Sensitivity: 0.737, Specificity: 0.824, PPV: 0.097, NPV: 0.992, Accuracy: 0.780	5-fold CV	To predict the onset of depression for easier and more effective treatment.
Sharma et al. [44] (2020)	Depression	Biomarkers and self-reported depression data	11,081 samples	XGBoost	Xgb.O Accuracy: 0.9729, B. Accuracy 0.9765, Precision:0.9548, Recall: 0.9987, F1-Score: 0.9762	CV	To cut the prolonged process of patient interviews and time cost.
Deshpande et al. [46] (2017)	Depression	Text (Twitter)	10,000 tweets	MNB SVM	MNB Precision: 0.836 Recall: 0.83 F1-Score: 0.8329 Accuracy: 83% SVM Precision: 0.804 Recall: 0.79 F1-Score: 0.7973 Accuracy: 79%	No Cross Validation	To detect depression by applying supervised learning algorithms on a text dataset.
Hilbert et al. [47] (2017)	Anxiety/ Depression	Image (MRI.)	19 GAD. 14 MD 24 Healthy controls	SVM	Case-classification: Accuracy: 90.10% Disorder-classification Accuracy: 67.46%	LOOCV	To prove the possibility of using biomarkers in the diagnosis of mental disorders.
Richter et al. [48] (2021)	Anxiety/ Depression	Clinical data Questionnaires	101 participants	RF	Anxiety/Depression/Mixed groups vs. control Specificity: 76.81%, Sensitivity: 69.66% Anxiety vs. Depression Specificity: 80.50%, Sensitivity: 66.46%	LOOCV	To provide a novel psychiatric diagnostic tool for differentiating between anxiety and depression patients.
Li et al. [49] (2020)	Bipolar disorder	MRI and Clinical evaluation	44 patients 36 controls	SVM	Accuracy: 87.5% Sensitivity: 86.4% Specificity: 88.9%	LOOCV	To differentiate patients with bipolar disorder from controls through the use of multimodal MRI data.
Li et al. [50] (2021)	Bipolar disorder/ first-episode psychosis (FEP.)	Image (sMRI)	89 FEP. 40 BD. 83 Healthy controls	CNN	Precision: 99.76% Recall: 99.74% F1-Score: 99.75% Accuracy: 99.72% AUC: 99.75% on the 3-way classification task	10-fold CV	To effectively increase the classification accuracy of mental disorders by extracting deep information from neuroimaging data.
Abaei and Osman [51] (2020)	Bipolar Disorder	Video	47 subjects 208 video recording	Hybrid CNN-LSTM model.	UAR: 60.67% Accuracy: 63.32%	RS	To discriminate between different levels of bipolar disorder through visual clues.
Rosellini et al. [54] (2018)	PTSD	Text (survey)	23,907 subjects	Super learner algorithm used on 39 individual algorithms.	AUC: 79.04	10-fold CV	To use machine learning in developing a post-earthquake PTSD risk score estimator.
Schultebraucks et al. [55] (2021)	PTSD	Clinical data	473 subjects	RF SVM	AUC: RF: 78% SVM: 88%	75%:25% RS	To determine if a pre-collected set of variables can be informative in the prediction of PTSD development over the course of time in active-duty army personnel.
Reece et al. [56] (2017)	Depression/ PTSD	Text (Twitter)	279,951 tweets from 204 users for depression/ 243,775 tweets from 174 users for PTSD	Various Supervised learning algorithms: 1200-tree RF classifier	Performance is shown in Table 1	5-fold CV	To forecast the onset of depression and PTSD among Twitter users.
Campbell et al. [57] (2019)	PTSD	Text (Survey)	2290 subjects	Decision tree analysis	Individual predictions in development samples: Sensitivity: 0.425 Specificity: 0.880	Independent Testing	To show how data from consecutive survey years can be used to create and validate an algorithm for the prediction of PTSD risks.
Gokten and Uyulan [58] (2021)	PTSD/ Depression	Clinical data	482 Children and adolescents	RF	AUC: Depression: 88.0% PTSD: 76.0%	10-fold CV	To determine the effect of various factors in the development of mental disorders.
Paul et al. [59] (2018)	Anorexia/Depression	Text (Reddit)	472 users 253,752 posts [60]	Ada Boost LR RF SVM	Overall performance: SVM on BOW: Precision: 0.97, Recall: 0.98, F-measure: 0.98. UMLS features: SVM: F-measure: 0.55 BOW and UMLS: Ada boost classifier: F-measure: 0.47	10-fold CV	To recognize anorexia in a timely manner in order to help professionals intervene.
Guo et al. [61] (2015)	Anorexia Nervosa	Genome genotyping data	3940 AN cases 9266 controls	LR SVM Gradient Boosted Trees	AUC: LR: 0.693 SVM: 0.691 Gradient Boosted Trees: 0.623	10-fold CV	To use genetic information in determining the risk factors of anorexia nervosa.
Ranganathan et al. [62] (2019)	Anorexia Nervosa	Text (Reddit)	472 Users [60]	i. Neural Machine Translator (Seq2Seq) ii. Traditional learning approach: SVM classifier with SGD optimization using TF-IDF	Overall Performance: Precision: 0.48 Recall: 0.26 F1-Score: 0.34 ERDE-50: 0.07	Independent Testing	To apply natural language processing methods to accomplish the detection and management of anorexia nervosa in its rudimentary stage.
López-Úbeda et al. [64] (2021)	Anorexia Nervosa	Text (Spanish Tweets)	5707 tweets [63]	i. Transfer learning methods: BETO M-BERT XLM ii. NN methods: LSTM BiLSTM CNN	Best performance (BETO): F1-Score: 94.1%	10-fold CV	To use machine learning classification algorithms to detect anorexia from Twitter comments in Spanish with a transfer learning technique.
Mikolas et al. [65] (2022)	ADHD	Clinical data	299 participants	SVM	Performance on 30 features: Accuracy: 66.1% Without demographic features: Accuracy: 65.1% Without missing data: Accuracy: 68.8%	10-fold CV	To appropriately distinguish ADHD in children or teenagers from a variety of other mental health issues.
Tan et al. [66] (2017)	ADHD	Image (fMRI)	265 subjects (NYU; ADHD-200 dataset [71])	SVM	Accuracy: 67.7%	10-fold CV (10 iterations)	To test if fMRI images can give additional information on brain volume abnormalities in ADHD patients that are not included in anatomical images, and hence may lead to a better classification model for the automatic diagnosis of ADHD.
Tachmazidis et al. [67] (2021)	ADHD	Questionnaires and Clinical data	69 patients	A hybrid model consisting of a Machine Learning and knowledge-based model	Accuracy: 95%	LOOCV	To find a way through which clinical information can create a decision tool to automate the process of diagnosis.
Peng et al. [68] (2013)	ADHD	Image (MRI)	55 patients 55 controls (Peking University; ADHD-200 dataset )	SVM-Linear SVM-RBF ELM learning algorithm	Accuracy: SVM-Linear: 84.73% SVM-RBF: 86.55% ELM: 90.18%	LOOCV	To establish a method for diagnosing ADHD that is automated, effective, quick, and accurate in order to address the shortcomings of traditional methods.
Yin et al. [69] (2022)	ADHD	Resting state fMRI	360 ADHD and TDC subjects	XGBoost	Differentiating ADHD from TDC; Accuracy: 77% (CV), 74.46% (IT) Predicting ADHD severity; *R*^2^: 0.2794 (CV), 0.156 (IT)	10-fold CV (10 iterations)	To determine if neural flexibility can serve as a biomarker to differentiate children with ADHD from typically developing children (TDC).
Liu et al. [70] (2020)	ADHD	Image (fMRI)	ADHD-200 dataset (Table 2)	CDAE-AdaDT model	Accuracy: 75.64%, Sensitivity: 76.92%, Specificity: 73.08%	No Cross Validation	To improve the result of ADHD classification in fMRI data.

Attention deficit hyperactivity disorder (ADHD), post-traumatic stress disorder (PTSD), term frequency–inverse document frequency (TF-IDF), support vector machine (SVM), logistic regression (LR), random forest (RF), neural network (NN), sequential minimal optimization (SMO), K-Star (KS), naïve Bayes (NB), Bayesian network (BN), extreme gradient boosting (XGBoost), convolutional neural networks (CNN), long-short-term memory (LSTM), stochastic gradient descent (SGD), extreme learning machine (ELM), convolutional denoising autoencoder, and adaptive boosting decision trees (CDAE-AdaDT model), area under the ROC curve (AUC), deep neural networks (DNN), functional magnetic resonance imaging (fMRI), magnetic resonance imaging (MRI), positive predictive value (PPV), negative predictive Value (NPV), balanced accuracy (B. Accuracy), multinomial naïve Bayes (MNB), early risk detection error (ERDE), General Psychology Corpus (GPC), traditionally developing children (TDC), cross-validation (CV: type not stated), cross-validation with dataset divided into 10 parts (10-fold CV), independent testing (IT), random split (RS: split proportion not stated), leave-one-out cross validation (LOOCV).

## 5. Analysis and Discussion

For this study, 32 articles were sourced primarily designed to aid in diagnosing mental illnesses. However, this study did not include ML or DL methods for treating the included mental diseases. The articles reviewed in this study were classified into six types of mental health conditions which include schizophrenia, depression, anxiety, bipolar disorder, post-traumatic stress disorder, anorexia nervosa, and attention deficit hyperactivity disorder. Twenty articles focused on the use of ML methods. Eight articles solely implemented DL approaches in their study, and four articles effectuated a combination of ML and DL approaches to attain better results.

It can be seen that over the last decade, research in the area of mental health diagnosis has slowly been turning towards development with DL algorithms. However, ML algorithms also played and still play a significant role in this research area, including LR, SVM, K-nearest neighbor, naïve Bayes, decision trees, and ensemble methods. SVM and RF appear to be the most commonly chosen classification methods, with over half of the 20 ML articles applying at least one of the two methods or the inclusion of both. SVM (SVM) and RF were compared by Birnbaum et al. [34], Jo et al. [35], Sau and Bhakta [37], Schultebraucks et al. [55], and Paul et al. [59] due to their ability to provide exemplary performance in terms of accuracy. Paul et al. [59] measured the performance of four different classifiers in terms of F-measure. From their findings, SVM performed better when validated using BOW and UMLS features separately; however, when combining BOW and UMLS features, the Ada Boost classifier outperformed other classifiers in terms of F-measure. Meanwhile, Sau and Bhakta showed that the Catboost classifier, when trained with 10-fold cross-validation, outperforms SVM (accuracy: 82.1%, precision: 80.7%) and RF (accuracy: 78.6%, precision: 80.7%), with a predictive accuracy and precision of 89.3% and 89.0%, respectively.

The application of diverse approaches used by [31,32,33,39,41,42,50,51,64] shows the wide possibilities of using DL methods for mental health diagnosis with good results. In the research carried out by Li et al. [50], their end-to-end CNN architecture showed excellent precision (99.76%), Recall (99.74%), F1-Score (99.75%), accuracy (99.72%) and AUC (99.75%) in a three-way classification task. Additionally, according to the analysis carried out by Srinivasagopalan et al. [31], their proposed DL technique showed high accuracy in the diagnosis of schizophrenia when compared to traditional ML approaches. All the articles which implemented DL methods included in this study showed at least an accuracy and F1-Score of 63.32% [51], and 63.50% [41], respectively.

Finally, the combination of DL and traditional ML models also shows encouraging accuracy results when trained on small datasets without overfitting [83]. Ranganathan et al. [62] employed RNN-LSTM and SVM classifiers in their research and discovered that their two-layer LSTM with normed-bahdanau attention outperformed the other models tested. With the open access ADHD-200 dataset, Peng et al. [68] and Liu et al. [70] implemented the combination of ML and DL models, with their performance ranging from 70 to 90%.

### 5.1. Datasets

Google has demonstrated significant success in training basic linear regression models on massive datasets throughout the years, proving that simple models can often outperform large models when trained on small datasets. However, the use of small data sizes in mental health research is common. Determining the smallness or largeness of data depends wholly on the project at hand, and many research results have been negatively impacted due to the low amount of training data. As can be observed in Table 3, sample datasets lower than 1000 subjects were used by [31,32,33,34,35,37,38,41,47,48,49,50,51,55,58,62,65,66,67,68,69,70]. Moreover, some of the reviewed studies implemented the use of datasets containing over 1000 subjects, such as [39,42,43,44,46,54,56,57,59,61,64].

### 5.2. Evaluation Metrics

Evaluation metrics are used to evaluate the quality of a statistical or ML model. Models may be assessed using various metrics, broadly classified as classification and regression metrics. Accuracy, area under the ROC curve (AUC), F1-Score, precision, mean absolute error, root mean squared error, Recall, sensitivity (true positive rate), and specificity (true negative rate) were utilized as assessment measures for the articles included in this study. It is strongly recommended that a separate dataset be used for performance evaluation instead of the same data used for model training or validation. This is because evaluating a model on untrained data allows you to determine whether or not the model is overfitting.

### 5.3. Challenges

We outlined some challenges in DL and ML approaches for mental health diagnosis for this review. First, according to Vabalas et al. [84], small sample sizes are prevalent in the mental health field due to the high expense of data collecting that requires human participation. Although many ML models may demonstrate resilience when trained on a limited sample size of data without sacrificing performance accuracy, the same cannot be said about DL models. When experimenting with DL models, extensive training data are often required since they allow researchers to comb parameter space while also allowing the model to generalize to avoid overfitting hazards.

Secondly, overfitting impacts model performance in deep learning. The problem of overfitting has been on the rise in recent research. This problem occurs when a model performs excellently on training data but generalizes poorly to unseen data. This could be due to the limited size of the training dataset, model complexity, or an imbalance in the training data. Although overfitting cannot be eliminated entirely, hyperparameters such as epochs, dropout, model regularization, activation function, as well as the number of hidden layers, could be tuned to reduce its effects. To mitigate this problem, it is also necessary to introduce independent test data, as they cause the model to generalize well to unseen data, thereby helping with the overfitting problem. Liu et al. [85] proved this as their experiment showed good performance after testing their model against an independent test dataset.

Thirdly, with the frequently varying mental health status of patients, and the close symptom-relatedness of some mental health conditions, one of the biggest challenges is the clear-cut diagnosis or prediction of these disorders over a long period. Researchers can look into developing effective models that detect different symptom intensities of the specified disorder in question and put into consideration the different scenarios in these disorders that change over time.

Finally, having access to meaningful, high-quality, large-scale data in the mental health sector is a significant challenge. This is owing to ethical and privacy concerns around subject recruitment, cost, and the nature of data collection, which frequently necessitates multi-disciplinary collaboration with healthcare specialists. Before retrieving data from individuals, further procedures might be taken to improve informed consent and user confidence. Additionally, building anonymous responsible mental health data repositories where people may freely submit information about their mental health disorders for research reasons could increase participants’ confidence.

## 6. Conclusions

Several papers on the diagnosis of seven distinct mental health conditions were reviewed to understand various machine learning (ML) and deep learning (DL) approaches that researchers have implemented in at least the last decade. This research considers that ML and DL technologies have both produced excellent outcomes when used to diagnose mental health disorders. The methodologies of ML and DL utilized by various researchers, as well as the databases used, have all been examined, and the challenges encountered are also outlined. Many approaches such as naïve Bayes, LSTM-RNN, logistic regression, support vector machines, random forest, neural networks, and others are being applied to discover patterns and hence diagnose mental illnesses. López-Úbeda et al. [64] proved that exploring other options such as transfer learning can also show excellent results. The efficiency and performance of various algorithms were also evaluated to determine which works best.

During the course of this study’s preparation, certain constraints were identified. Only four search databases (Google Scholar, PubMed, Scopus, and Web of Science) were used to collect data, and only articles published in English were included. Furthermore, the focus was limited to seven mental health diseases, restricting the understanding of additional mental health conditions in this field of research, such as autism spectrum disorder (ASD). We do not dispute that ASD is also an important mental health condition that should be considered. Therefore, we hope to focus more attention on it in future research.

This study demonstrates that ML alone can be valuable in understanding mental health issues. However, the development of DL methods suggests the possibility of predicting one disorder while also diagnosing others. When applied to visual modalities, DL structures can also aid in identifying the disorders mentioned in this review. A thorough investigation of additional data modalities, such as sensors, has been shown to be an effective method of identifying patients’ mental states. This is not to deny that various data preprocessing approaches can impact the performance accuracy of these models. As a result, it is recommended that researchers evaluate different ML and DL approaches to select a greater performance accuracy effectively.

To increase model performance, the challenges mentioned above can be taken into consideration. Researchers can look into sourcing more high-quality data and developing more explainable DL models that can improve model deployment in the real world. It is worth emphasizing that these systems are helpful for research since they provide access to standards that will assist future researchers in obtaining better findings, improve diagnostic accuracy, and possibly enhance professional decision-making when treating patients with these conditions.

## Figures and Tables

**Table 1:** Comparison results as shown by Reece et al. [56]. Cells marked N/A indicate unavailable metrics from previous studies.

Depression	MVR *µ*	DC *µ*	Daily *µ(σ)*	Weekly *µ(σ)*
Recall	0.510	0.614	0.518 (0.000)	0.521 (0.000)
Specificity	0.813	N/A	0.958 (0.000)	0.969 (0.000)
Precision	0.42	0.742	0.852 (0.000)	0.866 (0.000)
NPV	0.858	N/A	0.812 (0.000)	0.841 (0.000)
F1	0.461	0.672	0.644 (0.000)	0.651 (0.000)
**PTSD**	**TBA** *µ*	**NHC** *µ*	**Daily** *µ(σ)*	**Weekly** *µ(σ)*
Recall	0.249	0.82	0.683 (0.000)	0.658 (0.000)
Specificity	0.979	N/A	0.988 (0.000)	0.994 (0.000)
Precision	0.429	0.86	0.882 (0.000)	0.934 (0.000)
NPV	0.602	N/A	0.959 (0.000)	0.954 (0.000)
F1	0.315	0.84	0.769 (0.000)	0.772 (0.000)

## Data Availability

Not applicable.
